# Supplementary material for: Biodistribution and Pharmacokinectics of Liposomes and Exosomes in a Mouse Model of Sepsis
Source: Pharmaceutics. 2021 Mar 22;13(3):427. doi: 10.3390/pharmaceutics13030427 (PMC8004782; doi:10.3390/pharmaceutics13030427)
Supplement: Supplementary file 1 [file pharmaceutics-13-00427-s001.pdf]

pharmaceutics

MDPI

Supplementary Materials: Biodistribution and Pharmacokinectics of Liposomes and Exosomes in a Mouse Model of Sepsis

Amin Mirzaaghasi, Yunho Han, So Hee Ahn, Chulhee Choi* and Ji-Ho Park*

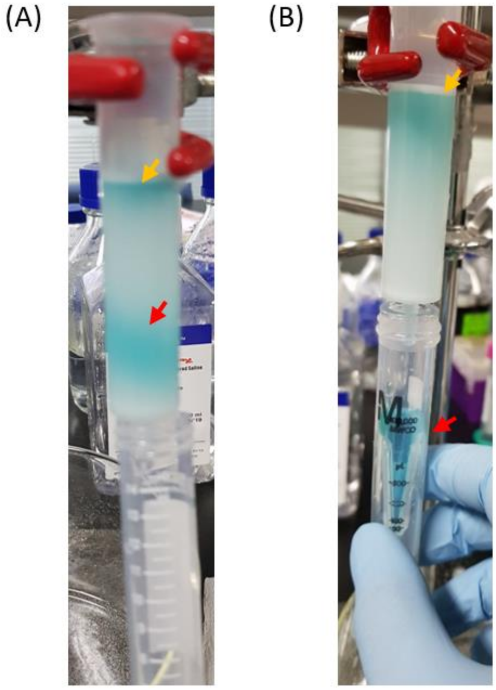

Figure S1. SEC method for removing free dyes**.** (A) SEC column before collecting exosomes. (B) SEC Column after collecting exosomes.

Publisher's Note: MDPI stays neutral with regard to jurisdictional claims in published maps and institutional affiliations.

Copyright: © 2021 by the author. Licensee MDPI, Basel, Switzerland. This article is an open access article distributed under the terms and conditions of the Creative Commons Attribution (CC BY) license (http://creativecommons.org/licenses/by/4.0/).

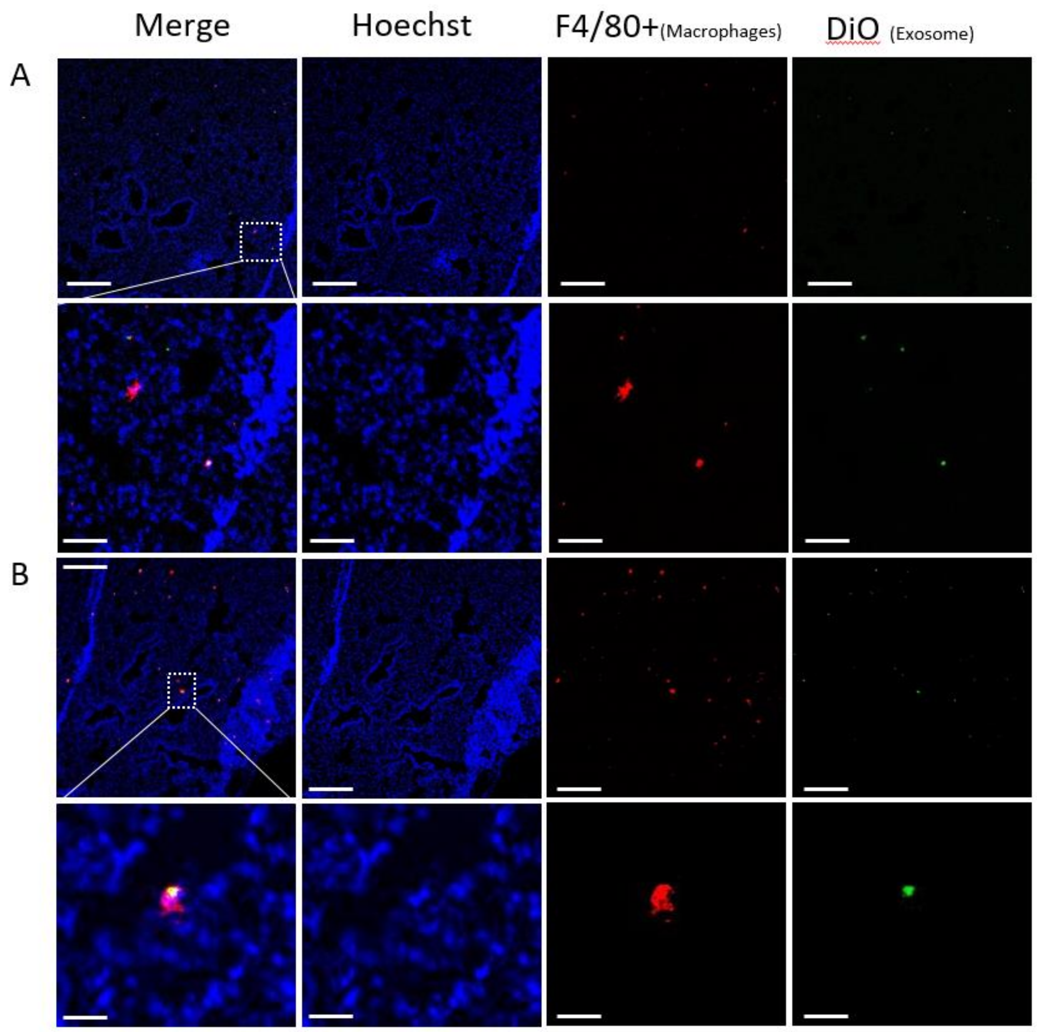

Figure S2. Confocal fluorescence microscopy of lung tissue sections after intravenous injection of exosomes. (A and B) Confocal fluorescence microscopic images of lung tissue sections obtained from healthy (A) and sepsis mouse (B) one hour after intravenous injection of DiO-labeled exosomes (Green). Macrophages and nuclei were stained with F4/80 (Red) and Hoechst 33342 (blue), respectively. Scale bar indicate 100 μm.
